# Activation of Sterol Regulatory Element Binding Protein and NLRP3 Inflammasome in Atherosclerotic Lesion Development in Diabetic Pigs

**DOI:** 10.1371/journal.pone.0067532

**Published:** 2013-06-25

**Authors:** Yu Li, Shanqin Xu, Bingbing Jiang, Richard A. Cohen, Mengwei Zang

**Affiliations:** Vascular Biology Section, Department of Medicine, Whitaker Cardiovascular Institute, Boston University School of Medicine, Boston, Massachusetts, United States of America; Baker IDI Heart and Diabetes Institute, Australia

## Abstract

**Background:**

Aberrantly elevated sterol regulatory element binding protein (SREBP), the lipogenic transcription factor, contributes to the development of fatty liver and insulin resistance in animals. Our recent studies have discovered that AMP-activated protein kinase (AMPK) phosphorylates SREBP at Ser-327 and inhibits its activity, represses SREBP-dependent lipogenesis, and thereby ameliorates hepatic steatosis and atherosclerosis in insulin-resistant LDLR^−/−^ mice. Chronic inflammation and activation of NLRP3 inflammasome have been implicated in atherosclerosis and fatty liver disease. However, whether SREBP is involved in vascular lipid accumulation and inflammation in atherosclerosis remains largely unknown.

**Principal Findings:**

The preclinical study with aortic pouch biopsy specimens from humans with atherosclerosis and diabetes shows intense immunostaining for SREBP-1 and the inflammatory marker VCAM-1 in atherosclerotic plaques. The cleavage processing of SREBP-1 and -2 and expression of their target genes are increased in the well-established porcine model of diabetes and atherosclerosis, which develops human-like, complex atherosclerotic plaques. Immunostaining analysis indicates an elevation in SREBP-1 that is primarily localized in endothelial cells and in infiltrated macrophages within fatty streaks, fibrous caps with necrotic cores, and cholesterol crystals in advanced lesions. Moreover, concomitant suppression of NAD-dependent deacetylase SIRT1 and AMPK is observed in atherosclerotic pigs, which leads to the proteolytic activation of SREBP-1 by diminishing the deacetylation and Ser-372 phosphorylation of SREBP-1. Aberrantly elevated NLRP3 inflammasome markers are evidenced by increased expression of inflammasome components including NLPR3, ASC, and IL-1β. The increase in SREBP-1 activity and IL-1β production in lesions is associated with vascular inflammation and endothelial dysfunction in atherosclerotic pig aorta, as demonstrated by the induction of NF-κB, VCAM-1, iNOS, and COX-2, as well as by the repression of eNOS.

**Conclusions:**

These translational studies provide *in vivo* evidence that the dysregulation of SIRT1-AMPK-SREBP and stimulation of NLRP3 inflammasome may contribute to vascular lipid deposition and inflammation in atherosclerosis.

## Introduction

Atherosclerosis, a chronic inflammatory disease, is the most common cause of cardiovascular death [1], [2]. Diabetes is a major independent risk factor for atherosclerotic cardiovascular disease [2]. A key early step in atherogenesis is lipid deposition within the arterial intima, which in turn promotes leukocyte recruitment, foam cell formation, endothelial cell activation, and vascular inflammation [1], [2]. Major clinical complications arise when atherosclerotic lesions evolve into complex and unstable forms, characterized by a thin fibrous cap and a large lipid-filled necrotic core [1]. While inflammation is thought to be a hallmark of advanced atherosclerosis [1], [2], the molecular mechanisms that link lipid sensing pathways to vascular inflammation in atherogenesis remain elusive.

Sterol regulatory element binding protein (SREBP), a key lipogenic transcription factor, resides as an inactive trans-membrane precursor in the ER. Once activated, SREBP is escorted to the Golgi where it is processed sequentially by two proteases to release the active fragment of SREBP [3]. The active mature form of SREBP enters the nucleus and activates the transcription of its target lipogenic genes encoding enzymes that are necessary for converting acetyl-CoA to fatty acids, triglyceride, and cholesterol under physiological conditions such as a refeeding state [3]. However, aberrantly elevated SREBP-dependent *de novo* lipogenesis contributes to the development of hepatic steatosis in insulin resistance [4], [5]. We have recently discovered that AMP-activated protein kinase (AMPK) directly phosphorylates SREBP-1 at Ser-372, represses the cleavage processing of SREBP-1, and suppresses the transcription of its own gene and targets *in vitro* and *in vivo* [6]. AMPK activation by polyphenols attenuates hepatic steatosis and ameliorates aortic atherosclerosis in insulin-resistant LDLR^−/−^ mice, at least in part, by suppressing the cleavage processing of SREBP-1 and SREBP-2 and lipid biosynthesis in the liver [6]. The function of SREBP on lipid homeostasis in the liver and adipose tissue as well as its deregulation on metabolic abnormalities in fatty liver disease and obesity are well characterized [3], [5]. Although non-alcoholic fatty liver disease and atherosclerotic disease in humans have common pathological features such as the deposition of excess lipids in the liver or on the vascular wall, little is known about the relationship between SREBP and vascular dysfunction in atherosclerosis. This translational study provides the first evidence for an elevation of SREBP in atherosclerotic lesions in humans with diabetes.

The nucleotide-binding domain, leucine-rich-containing family, pyrin domain-containing-3 (NLRP3) inflammasome has emerged as an important regulator of inflammation in metabolic disorders and atherosclerosis [7], [8]. The NLRP3 inflammasome, a cytosolic protein complex that consists of the regulatory subunit NLRP3, the adaptor apoptosis-associated speck-like protein (ASC, also known as pycard), and the effector caspase-1 [9], is activated by “endogenous danger signals”, such as the components released from necrotic cells [9], [10] and cholesterol crystals [7]. The stimulation of NLRP3 inflammasome triggers caspase-1 activation and subsequently promotes the cleavage processing and secretion of pro-inflammatory cytokine interleukin-1β (IL-1β) [9]. Recently, SREBP-1a has emerged to directly activate the transcription of the NLRP gene in macrophages, which can couple lipogenesis with the innate immune response in endotoxic shock [11]. However, it remains largely unknown whether dysregulation of SREBP is linked to vascular inflammasome in atherogenesis.

The present study is the first to show an elevation of SREBP-mediated lipotoxic signaling in atherosclerosis in pigs and humans. Our clinical study with aorta pouch biopsy specimens from patients with diabetes and coronary artery atherosclerosis shows intense immunostaining for SREBP-1 and vascular cell adhesion molecule-1 (VCAM-1), a key inflammatory marker, in atherosclerotic plaques. To gain direct insight into a novel vascular function of SREBP, we take advantage of a well-established porcine model of diabetes-accelerated atherosclerosis, in which plasma lipoprotein profiles with high levels of LDL in swine resemble those in humans, in contrast to high levels of HDL in mice [12]. This atherosclerotic pig model is also capable of developing human-like atherosclerotic plaques with variable and complicated lesion phenotypes [13]. Our results show that diabetic, atherosclerotic pigs display striking phenotypic hallmarks of atherosclerotic lesions such as a mixture of early fatty streaks and advanced lesions with fibrous caps and necrotic lipid cores. The cleavage processing of SREBP-1 and -2 and expression of their target lipogenesis genes are increased in the aorta of atherosclerotic pigs. The hyperactivation of SREBP-1 is likely attributed to integrated suppression of SIRT1 and AMPK and impairment of the deacetylation and phosphorylation of SREBP-1. Abnormal activation of SREBP-1 is correlated with enhanced vascular inflammatory response in atherosclerotic pig aorta, as evidenced by the upregulation of inflammasome markers including NLRP3, ASC, and IL-1β, as well as an elevation of proinflammatory mediators such as VCAM-1, nuclear factor-κB (NF-κB), inducible nitric oxide synthase (iNOS), and cyclooxygenase-2 (COX-2). These preclinical studies provide biochemical evidence that the dysregulation of SIRT1-AMPK-SREBP pathway and stimulation of NLRP3 inflammasome and IL-1β production may coordinately contribute to the initiation and progression of atherosclerosis.

## Materials and Methods

### Atherosclerotic artery specimens from humans with diabetes

Human aortic punch biopsies from patients with diabetes mellitus and coronary artery atherosclerosis at the time of coronary artery bypass surgery were obtained from a study published previously [14]. For Oil Red O staining, portions of fresh aortic tissues were mounted in OCT medium, snap-frozen on dry ice, and processed for cryosectioning. Portion of these tissues was rapidly fixed in 10% phosphate-buffered formalin acetate for immunohistochemistry as described previously [6], [15], [16]. Patients gave their written consent for their samples to be collected. Study of human specimens was approved by the Boston University Medical Center Institutional Review Board.

### The porcine model of diabetes-accelerated atherosclerosis

A well-established porcine model of diabetes and atherosclerosis was developed by Dr. Gerrity as described previously [13], which was evidenced by a 7-fold increase in fasting glucose levels (294±23 *vs.* 43±3 mg/dl) and an 8-fold elevation in plasma cholesterol levels (741±20 *vs.* 86±3 mg/dl). Briefly, diabetes was induced in 12-week-old male pigs (15–20 kg body weight) by the ear vein injection of streptozotocin (STZ, 50 mg/kg in 0.1 mol/L Na-citrate, pH 4.5, daily) for 3 consecutive days. The diabetic pigs were placed in a high cholesterol diet containing 1.5% cholesterol and 15% lard for 30 weeks. Non-diabetic control pigs were injected with a comparable volume of citrate buffer and placed on a Purina pig chow diet [13], [17]. When the pigs were sacrificed, aortic tissue samples were collected and kindly provided by Dr. Gerrity from a study published previously [13], [18] and stored at −80°C. For histology and immunohistochemistry, portions of the aortae were rapidly fixed in 10% phosphate-buffered formalin acetate at 4°C overnight, processed and embedded in paraffin, and sectioned as described previously [6], [15], [16].

### Histology and immunohistochemistry

For histological study, aortic sections from atherosclerotic pigs and humans were stained with hematoxylin and eosin (H&E) as well as with Oil Red O as described previously [6], [15], [16], [19]. For immunohistochemical studies, after removal of paraffin and rehydration, 5-µm thick adjacent aortic sections were treated with 10 mmol/L citric acid (pH 6.0) and heated in a microwave (2 min, 3 times at 700 W) to recover antigenicity. Nonspecific binding was blocked with 10% normal goat serum (Vector Laboratories, Burlingame, CA) in phosphate-buffered saline (PBS, pH 7.4) for 60 min. The sections were incubated with SREBP-1 antibody (sc-367, K10, 2 µg/mL), advanced glycation endproducts (AGE) antibody (RDI, 2 µg/mL), VCAM-1 antibody (sc-8304, 2 µg/mL), iNOS antibody (BIOMOL, 1∶100 dilution), COX-2 antibody (Cayman Chemical, 1∶500 dilution), endothelial nitric oxide synthase (eNOS) antibody (Transduction Laboratories, Cat. No. N30020, 5 µg/mL), or α-SM-actin (Sigma, 1∶200 dilution) in PBS with 1% BSA at 4°C overnight. The sections were washed and subsequently incubated at room temperature for 1 h with a biotinylated anti-rabbit or anti-mouse IgG secondary antibody (1∶500 dilution) using the Vectastain ABC kit. Positive immunoreactivity was visualized by red color of Vector® Red reaction product. Sections were counterstained with hematoxylin, cleared with xylene, and mounted. All positive staining was confirmed by ensuring that no staining occurred under the same conditions using nonimmune rabbit or mouse isotype control IgG (Vector). Staining images were captured and digitalized using an Olympus HC5000 digital camera attached to an Olympus microscope.

### Immunofluorescent double staining

Immunofluorescent double staining was performed as described previously [6]. All paraffin-embedded sections of control and atherosclerotic arteries were stained with MAC-2 antibody (ab53082, 1∶200 dilution) and detected using secondary antibody conjugated with Alexa fluor 555 (red). These sections stained with MAC-2 antibody were subsequently stained with either SREBP-1 antibody (sc-367, K10, 2 µg/mL) or IL-1β antibody (sc-78841, H-153, 1∶100 dilution) and detected using secondary antibody conjugated with Alexa fluor 488 (green). Finally, nuclei were counterstained with 4,6-diamidino-2-phenylindole (DAPI, blue). Overlapping images (yellow) of MAC-2 with either SREBP-1 or IL-1β indicates co-localization of SREBP-1 or IL-1β in inflamed macrophages of aortic plaques. The staining signals were specific inasmuch as incubation with non-immune IgG showed minimal detectable fluorescence under similar conditions. Images were captured under a fluorescence microscope (Nikon 80i Phase Contrast and Fluorescence Microscope, Japan).

### Immunoblotting analysis and SREBP-1 deacetylation experiments

Immunoblotting analysis was performed as described previously [6], [15], [19], [20]. The aortic tissues were homogenized in lysis buffer containing 20 mmol/L Tris-HCl, pH 8.0, 1% (vol/vol) NP-40, 150 mmol/L NaCl, 1 mmol/L EDTA, 1 mmol/L EGTA, 1 mmol/L sodium orthovanadate, 25 mmol/L β-glycerolphosphote, 1 mmol/L dithiothreitol, 1 mmol/L phenylmethylsulfonyl fluoride, 2 µg/mL aprotinin, 2 µg/mL leupeptin, and 1 µg/mL pepstatin, and incubated on ice for 1 h. For immunoblotting analysis of SREBP proteins with a modification protocol, the total tissue lysates were subjected to sonication on ice for 20 seconds with the Sonifier 250 (Branson; output 2, duty cycle 20%), followed by centrifugation at 14,000 rpm for 10 min at 4°C. Protein concentrations in supernatants were measured using a Bio-Rad protein assay kit, and total tissue lysates (100 µg proteins) were resolved by 8% SDS-PAGE and immunoblotted with specific antibodies. Rabbit polyclonal antibodies against phospho-Thr172 AMPKα (Cat. No. 2531), phospho-Ser-372 SREBP-1c (Cat. No. 9874), acetyl-Lys-382 p53 (Cat. No. 2525), acetyl-Lys-9 histone H3 (Cat. No. 9671), and acetylated-lysine (Cat. No. 9441) as well as rabbit polyclonal antibodies against AMPKα (Cat. No. 2532) and acetyl-CoA carboxylase (ACC, Cat. No. 3662) were obtained from Cell Signaling Technology (Beverly, MA) and used in this study. Rabbit polyclonal antibodies against phospho-Ser79 ACC (Cat. No. 07-303) and the NAD^+^-dependent deacetylase SIRT1 (Cat. No. 07-131) from Millipore (Lake Placid, NY) were also used. The specific band intensity was quantified by densitometric analysis with ImageJ software (http://rsb.info.nih.gov/ij/), normalized to that of loading control proteins, and expressed as relative levels to control pigs. For SREBP-1 deacetylation assays, SREBP-1 protein in total tissue lysates was immunoprecipitated with SREBP-1 antibody and Protein A/G-Sepharose beads at 4°C overnight. The precipitates were washed three times with ice-cold lysis buffer and twice with wash buffer (25 mmol/L Tris-HCl, pH 7.5, 50 mmol/L NaCl, 5 mmol/L MgCl_2_, 1 mmol/L dithiothreitol). The acetylation of SREBP-1 was subsequently detected by immunoblotting with an acetylated-lysine specific antibody.

### Quantitative real-time PCR

The aortic tissues were homogenized in TRIzol Reagent (Invitrogen) according to the manufacturer's protocol. Total RNA was reversely transcribed to cDNA by SuperScriptII reverse transcriptase (Invitrogen) using Oligo dT according to the manufacturer's protocol. The resulting cDNA was subjected to real-time PCR with gene-specific primers in the presence of SYBR Green PCR master mix (Applied Biosystems) using StepOnePlus Real-Time PCR System (Applied Biosystems) as described previously [6], [15]. The specificity of the PCR amplification was verified by melting curve analysis of the final products and by running products on an agarose gel. Data were analyzed using the ΔΔCt (cycle threshold) method. The mRNA levels of genes were normalized to those of β-actin and presented as relative levels to control pigs. Primers were designed using Primer3 (v. 0.4.0), and the following primers were utilized:

SREBP-1a, GGCCGAGCCATGCGAGCT (F) and TTGTTGATGAGCTGAAGCATGT (R);

SREBP-2, GCTGTGCGTGCTCACTTTAC(F) and TCTGCAGGTGTGGAAGACAG (R);

SCD1, ACCTGGCTGGTAAACAGTGC (F) and TAGTTGTGGAAGCCCTCACC (R);

GPAT, TGAAGACAGCGATTTTGGTG (F) and TGCAGGAAGGTGATGAACTG (R);

HMGCS, AAGTGGGCACTGAGACCATC (F) and TCAGTGTTGCCTGAGTCCTG (R);

NLRP3, CCTTCAGGCTGATTCAGGAG (F) and GACTCTTGCCGCTATCCATC (R);

ASC (Pycard), ACAACAAACCAGCACTGCAC (F) and CTGCCTGGTACTGCTCTTCC (R);

Caspase-1, GTCCTCGAACTCTCCACAGG (F) and GAAGACGCAGGCTTAACTGG (R);

IL-1β, CCTTGAAACGTGCAATGATG (F) and TTCAAGTCCCCTGTGAGGAG (R);

NF-κB, AACACCGCATAAACCAAAGC (F) and GTTCCTCTGAGCACCTCTGG (R);

β-actin, TTCCAGCAGATGTGGATCAG (F) and AGCCATGCCAATCTCATCTC (R).

### Statistical Analysis

Statistical analysis was performed using Student's t test with GraphPad Prism v5.0 software. Data are expressed as means ± standard error (S.E.M). An asterisk indicates statistical significant difference at P<0.05.

## Results

### Elevated SREBP-1 and inflammatory response are evident in aortic atherosclerotic lesions in humans with diabetes

**Figure 1:**
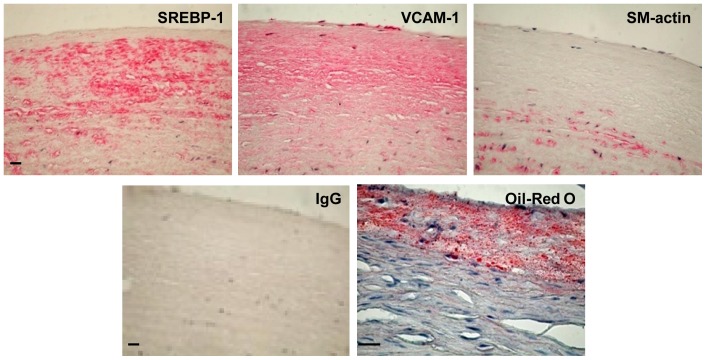
Positive immunostaining of SREBP-1 and VCAM-1, an important vascular inflammatory marker, in aortic sections of atherosclerotic lesions in humans with diabetes. Representative Oil Red O staining and immunostaining for SREBP-1, VCAM-1, and α-SM-actin in aortic sections of the atheromatous plaque in humans are shown (scale bars: 100 µm). Aortic sections shown were serial but not always consecutive. Positive immunostaining appeared red color. Positive staining of SREBP-1 was present primarily in subendothelial intimal lesions and lipid-rich macrophage plaque areas that were positively stained by Oil Red O. The distribution of positive staining for SREBP-1 was similar to that of VCAM-1, except VCAM-1 staining was much diffuse in lesions. The minimal staining of aortic sections in human atherosclerosis was present with the negative control staining by the incubation with non-immuno IgG substituted for the primary antibody.

To gain insight into the clinical relevance of SREBP-1 to vascular inflammation in human atherosclerosis, aortic pouch biopsy specimens were obtained from patients with diabetes and coronary artery atherosclerosis and immunostained for SREBP-1, α-smooth muscle actin (α-SM-actin), and VCAM-1, a critical endothelial-leukocyte adhesion molecule in atherogenesis [6], [16]. As shown in Fig. 1, the immunoreactivity for SREBP-1 was predominantly overlapped with atherosclerotic plaques containing lipid-laden macrophages that were positively stained by Oil Red O. Compared with minimal immunostaining of aortic sections with non-immuno IgG, the intense staining for SREBP-1 was primarily colocalized with VCAM-1-positive cells and macrophage-rich areas of the plaques, but to a lesser degree, in the lesions where smooth muscle cells were strongly stained with α-SM-actin. These data suggest a previously unrecognized relationship between SREBP-1 and increased adhesion molecules at early/intermediate stages of atherosclerotic plaques in diabetic patients.

### Characterization of aortic atherosclerotic lesions in a well-characterized pig model of diabetes and atherosclerosis

**Figure 2:**
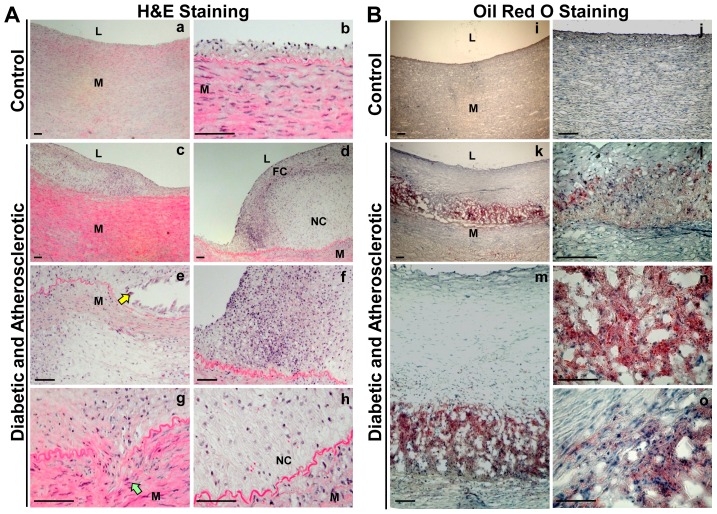
Characterization of human-like, complicated atherosclerotic phenotypes in the well-established porcine model of diabetes-accelerated atherosclerosis. Histopathologic characteristics of early/intermediate lesions and advanced plaques in diabetic and atherosclerotic pigs. Representative H&E staining (**A**) and Oil Red O staining (**B**) of aortic sections of control pigs (**a,b**, **i** and **j**) and diabetic, atherosclerotic pigs (**c–h** and **k–o**) are shown (scale bars, 100 µm). H&E-staining (**d–h**) showed a human-like fibroatheroma with a fibrous cap at the shoulder and a necrotic lipid core at the base of lesion (L = lumen, FC = fibrous cap, NC = necrotic lipid core, and M = media). Note the clear space (yellow arrow) which represents an area of cholesterol crystals removed during tissue processing (**e**) and smooth muscle cell migration from the media to the intima (green arrow) (**g**). The advanced plaques have necrotic lipid cores containing large numbers of cholesterol crystals (**m**, **n** and **o**).

Because of the limitation of aortic samples from diabetic, atherosclerotic patients, we took advantage of a well-established porcine model of diabetes-accelerated atherosclerosis [13], which was characterized by a mixture of early-to-mid stage lesions and advanced plaques (Fig. 2). Compared with control pigs, the early stage lesions were characterized by a series of events that include increased intimal thickening with lipid deposit, lipid accumulation preceding macrophage infiltration, and macrophage-derived foam cell formation (Fig. 2c). Advanced aortic lesions, such as a fibroatheroma, displayed a well-developed fibrous cap infiltrated by foamy macrophages overlying a necrotic lipid core at the base of advanced lesions (Fig. 2d–h). The fibrous cap in atherosclerotic plaques contained smooth muscle cells, which covered a mixture of lipid-laden foam cells, lipids and debris. The formation of more lipid-laden foam cells potentially promoted the migration and proliferation of smooth muscle cells from the media into the intima, resulting in plaque progression. The necrotic core formation represented the accumulation of lipids and the process of macrophage apoptosis and necrosis (Fig. 2e, h and k). The formation of cholesterol clefts and foam cells in subendothelial regions and the thickening of the arterial wall act as a part of the atheromatous process, which were visualized by Oil Red O staining of aortic sections in diabetic pigs (Fig. 2k–o), but not seen in control pigs (Fig. 2i and j). These pathological changes were consistent with previous studies [13] and similar to that observed in human atherosclerosis [21].

### The proteolytic processing of SREBP and expression of its target genes are increased in the aorta of diabetic, atherosclerotic pigs

**Figure 3:**
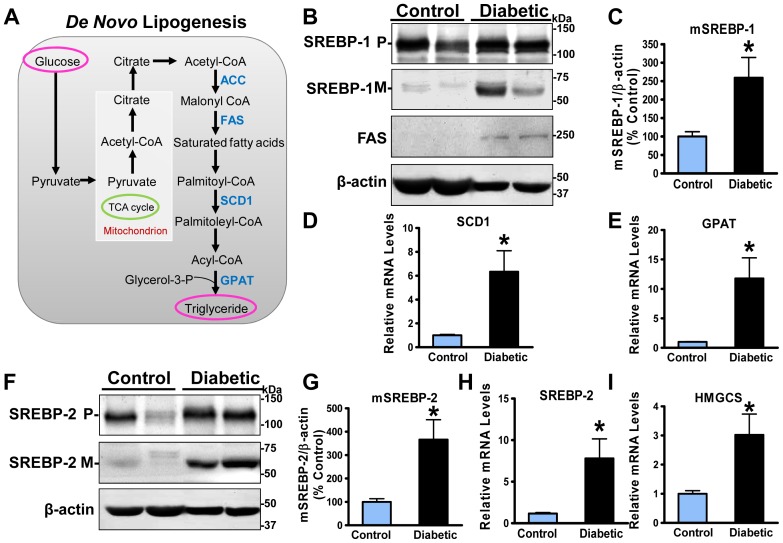
The cleavage processing of SREBP-1 and -2 as well as expression of their target genes are enhanced in the aorta of diabetic, atherosclerotic pigs. **A**. The transcriptional regulation of *de novo* lipogenic enzymes (blue color) by SREBP-1. **B**. The levels of mature, active form of SREBP-1 and expression of its target lipogenic enzyme, fatty acid synthase (FAS), are increased in the aorta of diabetic and atherosclerotic pigs. Total aortic lysates (100 µg proteins) were resolved by 8% SDS-PAGE and immunoblotted with the specific antibody to recognize both precursor (**P**, ∼125 kDa) and active mature (**M**, ∼68 kDa) forms of SREBP-1. Representative immunoblots of aortic tissues from two pigs in each group are shown. **C**. Densitometric quantification of the mature form of SREBP-1 is normalized to that of β-actin and expressed as relative levels to control pigs. **D** and **E**. The transcription of SREBP-1 target genes is elevated in the aorta of diabetic, atherosclerotic pigs. Total RNA was isolated from pig aorta, and mRNA levels of genes encoding stearoyl-CoA desaturase1 (SCD1) (**D**) and glycerol-3-phosphate acyltransferase (GPAT) (**E**) were determined by real-time PCR. **F** and **G**. The levels of the cleaved form of SREBP-2 protein were quantified by densitometry, normalized to those of β-actin, and expressed as relative levels to normal pigs. **H** and **I**. The mRNA amounts of SREBP-2 and its target gene, HMGCoA synthase (HMGCS), are elevated in atherosclerotic pigs. The mRNA levels of SREBP-2 and cholestrogenic genes were determined by real-time PCR, normalized to those of β-actin, and presented as relative levels to control pigs. Data were presented as the mean ± S.E.M., n = 3, *P<0.05, vs control pigs.

Because our studies and others have indicated that SREBP-1 plays a role in the regulation of *de novo* lipogenesis induced by sustained hyperglycemia in hepatocytes [6], [22], we hypothesized that the development of complex atherosclerotic lesions might be partially ascribed to increased SREBP activity in the arterial wall *in vivo*. To test this possibility, the proteolytic processing of SREBP precursor was assessed by an immunoblot analysis of the precursor (∼125 kDa) and cleavage forms (∼68 kDa) of SREBP [6]. Strikingly, the activated mature form of SREBP-1 was increased ∼2-fold in the aorta of atherosclerotic pigs as compared to that of normal pigs, whereas the membrane-bound SREBP-1 precursor was only slightly increased (Fig. 3B), suggesting enhanced cleavage processing of SREBP-1 in the atherosclerotic pig aorta. To further determine whether the accumulation of mature SREBP-1 is functionally relevant to the stimulation of *de novo* lipogenic genes, the transcription of SREBP-1 target genes was assessed by real-time PCR. The mRNA amounts of fatty acid synthase (FAS) and stearoyl CoA desaturase1 (SCD1), the key enzymes for fatty acid and triglyceride synthesis, were significantly increased in the aorta of atherosclerotic pigs, consistent with the elevated mature SREBP-1 (Fig. 3C–D). The mRNA levels of glycerol-3-phosphate acyltransferase (GPAT) were also robustly elevated 10-fold (Fig. 3E), comparable to those of lipogenic genes including SREBP-1, FAS, and SCD1. Taken together with strong staining of SREBP-1 in human atherosclerosis (Fig. 1), these results suggest that the proteolytic activation of SREBP-1 and its associated lipogenic process may contribute to lipid accumulation and deposition in atherosclerotic aorta.

We next examined the processed active form of SREBP-2 that governs the transcriptional regulation of its target genes required for cholesterol synthesis [3]. Like the induction of mature SREBP-1, the cleaved form of SREBP-2 was increased 3.7-fold and correspondingly accompanied by a moderate increase in SREBP-2 precursor (Fig. 3F and G), which is possibly due to an increase in the feed-forward regulation by the binding of active SREBP-2 to the SRE motif on its own promoters [3], [5], [6]. In support of this notion, mRNA levels of SREBP-2 and its target gene HMGCoA synthase (HMGCS), the rate-limiting enzyme in cholesterol biosynthesis, were elevated 8-fold and 3-fold, respectively (Fig. 3H and I). Taken together, the cleavage of SREBP-1 and -2 and expression of their key target genes involving triglyceride and cholesterol synthesis are locally enhanced in the vascular wall of atherosclerotic pigs, which likely explains an increased cholesterol synthesis in the aorta of atherosclerotic pigs seen in an earlier studies [13].

### Inhibition of AMPK activity is functionally relevant to the reduction of SREBP-1 phosphorylation at Ser-372 in the aorta of diabetic, atherosclerotic pigs

**Figure 4:**
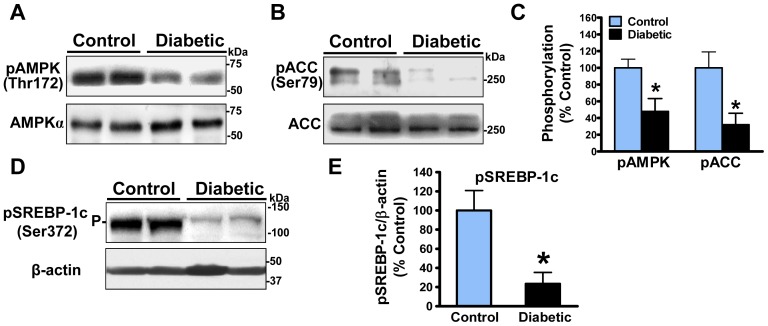
Inhibition of AMPK leads to impaired Ser-372 phosphorylation of SREBP-1 in the aorta of diabetic, atherosclerotic pigs. **A** and **B**. Repression of AMPK occurs in the aorta of diabetic and atherosclerotic pigs. Representative immunoblots for the phosphorylation of AMPKα at Thr-172 and acetyl-CoA carboxylase (ACC) at Ser-79, the well-known downstream target of AMPK, as well as the equal expression of AMPKα and ACC are shown. **C**. Densitometric quantification of the phosphorylation of AMPKα or ACC is normalized to that of endogenous AMPKα or ACC, respectively, and expressed as relative phosphorylation levels to normal pigs. **D** and **E**. Ser-372 phosphorylation of SREBP-1c precursor, a novel substrate of AMPK, is largely reduced in the aorta of diabetic and atherosclerotic pigs. Data were presented as the mean ± S.E.M., n = 3, *P<0.05, vs control pigs.

We have recently discovered that AMPK specifically phosphorylates SREBP-1 at Ser-372, which in turn inhibits its processing, nuclear translocation, and auto-regulation, leading to decreased *de novo* lipogenic process in hepatocytes [6]. Conversely, suppression of AMPK results in impaired phosphorylation of SREBP-1 and thereby increases the cleavage processing of SREBP-1 in the liver of obesity-induced insulin resistant mice [6]. To elucidate the molecular mechanisms underlying SREBP-1 activation in atherosclerotic lesions, a possible role of AMPK and its phosphorylation of a new substrate SREBP in the development of atherosclerosis were assessed by immunoblotting analysis. The phosphorylation of AMPK at Thr-172, which is required for AMPK activation [19], was dramatically declined by ∼40% in diabetic pig aorta, without affecting expression of endogenous AMPKα, compared to normal pig aorta (Fig. 4A–C). Likewise, suppression of AMPK activity was confirmed by a ∼70% reduction in phosphorylation of ACC at Ser-79, a well-known substrate of AMPK (Fig. 4B and C). These results are consistent with the inhibitory effect of hyperglycemia on hepatic AMPK activity *in vitro* [6], [19], [20] and in STZ-induced type 1 diabetic mice *in vivo* [19]. Interestingly, the phosphorylation of SREBP-1c precursor at Ser-372, a specific phosphorylation site of AMPK, was also reduced by approximately 70% (Fig. 4D and E), which was correlated well with the repression of AMPK phosphorylation (Fig. 4A and B). Our findings suggest that the pronounced defect in AMPK-mediated phosphorylation of SREBP-1 may be partially responsible for the proteolytic activation of SREBP-1 during atherosclerotic progression.

### The reduction of deacetylation of SREBP-1 is associated with suppression of SIRT1 in the aorta of diabetic, atherosclerotic pigs

**Figure 5:**
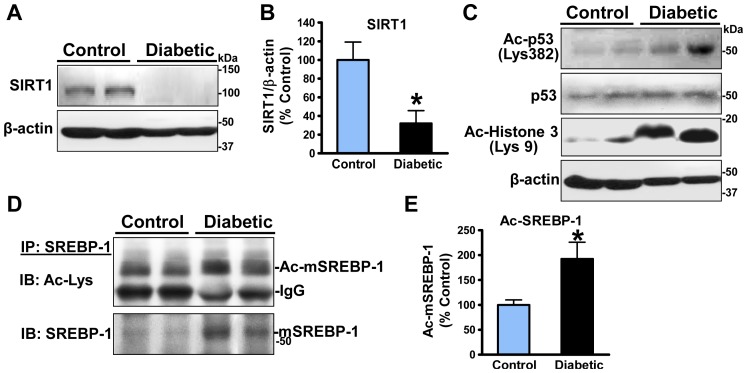
Downregulation of SIRT1 results in decreased deacetylation of the mature form SREBP-1 in the aorta of diabetic, atherosclerotic pigs. **A** and **B**. Expression of endogenous SIRT1 is remarkably reduced in the aorta of diabetic and atherosclerotic pigs. The protein levels of SIRT1 were normalized to those of β-actin and presented as relative levels to control pigs. **C**. The NAD^+^-dependent SIRT1 deacetylase activity, as reflected by deacetylation of p53 at Lys-382 and histone H3 at Lys-9, is diminished in the aorta of diabetic and atherosclerotic pigs. **D** and **E**. Deacetylation of mature SREBP-1 is reduced in atherosclerotic pigs. When endogenous SREBP-1 was immunoprecipitated with SREBP-1 antibody, the mature SREBP-1 in immunoprecipitates was assessed by immunoblots with an acetylated-lysine antibody and SREBP-1 antibody, respectively. Data were presented as the mean ± S.E.M., n = 3, *P<0.05, vs control pigs.

Weinberg's group was the first to identify that SIRT1 functions as an NAD-dependent deacetylase of p53 [23]. The deacetylation of p53 by SIRT1 was demonstrated as indicative of cellular deacetylase activity of SIRT1 [24]–[28]. The Lys-382 of p53 has further been identified as a specific deacetylation site of SIRT1 and detected by immunoblots with anti-acetylated p53 Lys-382 antibody [26]. Our recent studies indicate that overexpression of SIRT1 decreases the acetylation of p53 at Lys-382 in the liver of obese, insulin resistant mice [15]. Other studies show that SIRT1 decreases histone H3 at Lys-9 in ethanol-induced fatty liver in mice [29]. Because inhibition of SIRT1 is causally implicated in endothelial cell apoptosis in the atherosclerosis-prone aortic arch of high-fat diet-induced insulin resistant mice [30], expression and activity of SIRT1 were assessed in the aorta of atherosclerotic pigs. As shown in Fig. 5A and B, expression of SIRT1 was decreased by approximately 70% in the diabetic pig aorta, similar to the reduction of SIRT1 activity in diabetic mouse aorta [30]. Decreased SIRT1 activity was confirmed by a remarkable increase in the acetylation of p53 at Lys-382, accompanied by an elevation in the acetylation of histone H3 at Lys-9 (Fig. 5C). The impaired deacetylation of p53 or histone H3 was well correlated with a reduction of SIRT1 in atherosclerotic pig aorta, suggesting that defective SIRT1 activity is implicated in atherogenesis and vascular injury. Because SIRT1 is thought to negatively regulate SREBP-1 via a mechanism involving the direct deacetylation and degradation of the nuclear form of SREBP-1 [31]–[33], we speculated that defective SIRT1 might modulate SREBP-1 activity in atherosclerotic pigs. To this end, *in vivo* acetylation assays revealed that in parallel to the reduced SIRT1 activity, the mature form of SREBP-1 was hyperacetylated in atherosclerotic pig aorta, even though the basal acetylation of endogenous SREBP-1 was detectable in control pig aorta (Fig. 5D and E). Notably, increased cleaved form of SREBP-1 in atherosclerotic pig aorta was also observed in immunoprecipitates (Fig. 5D), similar to that seen in total cell lysates (Fig. 3B). These results suggest that the pro-atherogenic effect of SIRT1 dysfunction probably act through the inhibition of SREBP-1 deacetylation and stimulation of SREBP-1activity.

### The NLRP3 inflammasome markers are enhanced in the aorta of diabetic, atherosclerotic pigs

**Figure 6:**
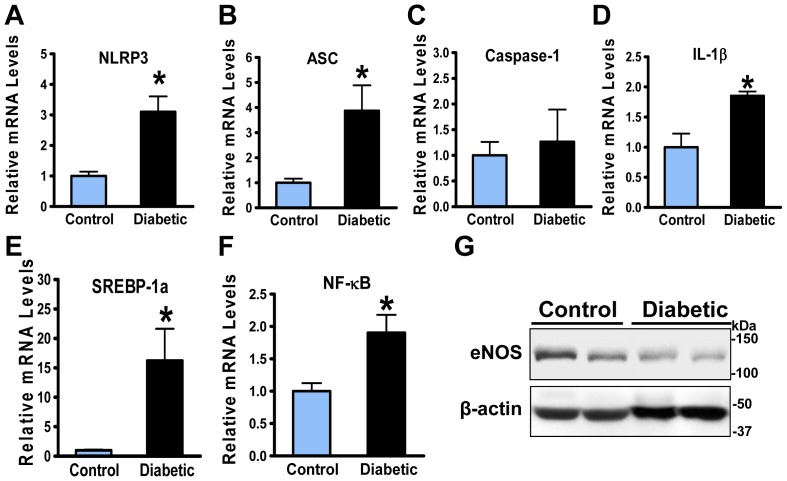
NLRP3 inflammasome and inflammatory response are increased in the aorta of diabetic, atherosclerotic pigs. **A–C**. expression of NLRP3 and apoptosis-associated speck-like protein (ASC) is increased in the aorta of diabetic, atherosclerotic pigs. **D**. The mRNA levels of proinflammatory cytokine interlucin-1 β (IL-1β) are increased in the aorta of diabetic, atherosclerotic pigs. **E** and **F**. The mRNA amounts of SREBP-1a and NF-κB are elevated in diabetic, atherosclerotic pigs. **G**. The expression of eNOS, indicative of endothelial function, is reduced in diabetic, atherosclerotic pigs. Data were presented as the mean ± S.E.M., n = 3, *P<0.05, vs control pigs.

NLRP3 inflammasome has emerged to regulate the processing of proinflammatory cytokines such as IL-1β [9]. Increased NLRP3 inflammasome components are implicated in the pathology of obese individuals with type 2 diabetes [8] and atherosclerosis [7]. A possible role of NLRP3 inflammasome in atherosclerotic pig aorta was assessed by determining gene expression of inflammasome components including NLRP3, ASC, caspase-1, and IL-1β. The mRNA amounts of NLRP3 and ASC were 3- to 4-fold higher in atherosclerotic pig aorta than those in control pig aorta, but no significant difference in caspase-1 was noted between two groups. The mRNA levels of IL-1β were elevated 2- to 3-fold, comparable to those of NLRP3 and ASC in atherosclerotic pig aorta (Fig. 6A–D). Taken together, the gene expression of NLRP3 inflammasome and IL-1β is increased during atherosclerotic progress.

### Cell types and distribution of SREBP-1 expression in early and advanced atherosclerotic lesions of diabetic pigs

**Figure 7:**
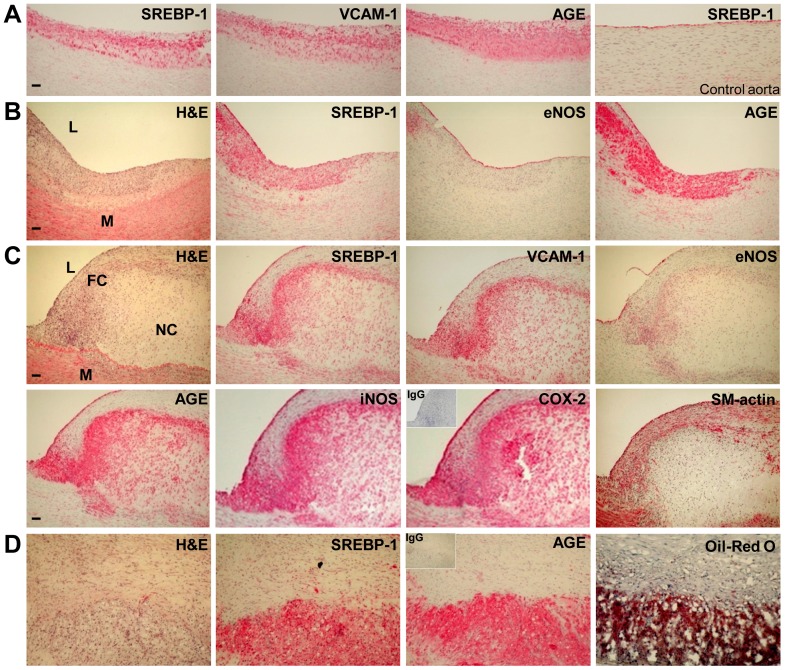
Immunostaining for SREBP-1 and vascular inflammatory mediators in a mixture of early/intermediate stage lesions and advanced plaques of diabetic, atherosclerotic pigs. A representative immunohistochemical staining of adjacent aortic sections of normal pigs and diabetic, atherosclerotic pigs is shown (scale bars: 100 µm). Positive immunostaining appeared red color. Immunostaining for SREBP-1 (a lipogenic marker) was located primarily in endothelial cells and in macrophage infiltration areas of fatty streaks (**A** and **B**), the inflamed fibrous cap with the necrotic lipid core (L = lumen, M = media, FC = fibrous cap, and NC = necrotic lipid core) (**C**), and lipid-laden foam cells and large numbers of cholesterol crystals under subendothelial lesion areas that were strongly stained by Oil Red O (**D**). Immunostaining for eNOS (an endothelial cell marker) showed impaired endothelium integrity in atherosclerotic lesions (**C**). Immunostaining for α-smooth muscle actin (α-SM-actin) displayed intense staining in the fibrous cap, indicating the migration and proliferation of SMCs into the intimal lesions (**C**). Immunostaining for VCAM-1, iNOS, and COX-2 (vascular inflammatory markers) displayed a pattern similar to SREBP-1 distribution in atherosclerotic lesions (**C**). Immunostaining for AGE (advanced glycation end products, a glucotoxic marker) showed a pattern similar to that of SREBP-1, except the staining of AGE was much intense in endothelial cells of the fibrous cap and in the necrotic plaque of advanced lesions (**C**). Positive staining for SREBP-1, VCAM-1, iNOS, COX-2, or AGE was also localized in most of endothelial cells and lipid-laden macrophages and in some of α-SM-actin-expressing SMCs in advanced plaques (**C**). In contrast, positive staining for SREBP-1 was only detected in endothelial cells of normal pigs (**A**). The minimal staining for VCAM-1, iNOS, COX-2, or AGE was present in normal pigs (data not shown). The *inset* images in the top left demonstrate the negative control staining by the incubation with non-immuno IgG substituted for the primary antibody (**C–D**).

To assess the potentially detrimental effects of SREBP-1 on vascular functions, cell types and expression distribution of SREBP-1, along with eNOS and α-SM-actin, were determined by immunohistochemical staining of adjacent aortic sections at various stages of plaques. As shown in Fig. 7, SREBP-1 was only detectable in endothelial cells (ECs), but not in other vascular cells, in the aorta of normal pigs (Fig. 7A). In contrast, immunoreactivity for SREBP-1 was remarkably intense on the endothelial layers in both early and advanced lesions of atherosclerotic pigs. The intense staining for SREBP-1 was also localized particularly in lipid-laden macrophages within atherosclerotic lesions that were strongly stained by Oil Red O (Fig. 7D), suggesting that increased SREBP-1 may facilitate efficient enzymes coupling to macrophage lipid synthesis in lesion areas. Infiltration of macrophages into the subendothelial space is thought to be a key step of atherogenesis [1], [2]. Cholesterol crystals are also recognized as a hallmark of atherosclerotic lesions in humans, and their appearance assists the histopathological classification of advanced atherosclerotic lesions [7], [34]. Strongly positive staining for SREBP-1 was noted extending into subendothelial lesions and overlapping with cholesterol clefts in necrotic lipid cores (Fig. 7D). Furthermore, the distribution of SREBP-1-positive cells in early and advance lesions was overlapped with that of endothelial cells that were positively stained by eNOS. Increased SREBP-1 immunoreactivity was also observed in some but not all of smooth muscle cells in the fibrous cap, which are positively stained by α-SM-actin. Positive staining for SREBP-1 was present, to a lesser extent, in necrotic cores of advanced lesions (Fig. 7C). Consistent with strongly positive staining for SREBP-1 in human atherosclerosis (Fig. 1), positive staining for SREBP-1 is primarily localized in multiple cell types in lipid-rich plaques of the diabetic, atherosclerotic pigs.

### Vascular inflammatory markers are increased during the development of complex lesions in diabetic, atherosclerotic pigs

The expression of vascular inflammatory markers was assessed to further determine whether the elevation of SREBP-1 and NLRP3- and ASC-mediated production of IL-1β is pathologically relevant to vascular inflammatory process and endothelial dysfunction in atherosclerosis. As shown in Fig. 6F and G, expression of NF-κB was increased, and expression of eNOS was declined in atherosclerotic pig aorta. The minimal immunostaining for VCAM-1, iNOS, and COX-2 was present in the aorta of non-diabetic pigs (data not shown). In contract, immunoreactivity for VCAM-1 was present in lipid-laden macrophage regions within fatty streaks predisposed to atherosclerosis, as was previously observed in aortic atherosclerotic plaques of STZ-induced type 1 diabetic ApoE^−/−^mice [16] and high-fat diet-induced type 2 diabetic LDLR^−/−^ mice [6]. Positive staining for VCAM-1 was significantly intense in eNOS-positive endothelial cells and scattered in α-SM-actin-positive cells of the fibrous cap (Fig. 7C). Moreover, immunohistochemical analysis showed that the staining intensity of iNOS and COX-2, NF-κB-dependent genes, was primarily located in the endothelium of intimal lesions and within the fibrous lesions (Fig. 7C). Notably, staining of iNOS and COX-2 was overlapped with that of VCAM-1 in a staining pattern similar to the SREBP-1 distribution. Furthermore, positive staining for advanced glycation end-products (AGE), a diabetes-specific inflammatory and oxidant activator [35], was similar to that of vascular inflammatory mediators and SREBP-1 within lesions (Fig. 7A–D). Because hyperglycemia-induced accumulation of AGE also promotes VCAM-1-dependent recruitment of leukocytes [35], our data strongly suggest that the synergistic effects of glucotoxicity and lipotoxicity potentially contribute to macrophage infiltration, endothelial dysfunction, and smooth muscle cell proliferation within fibrous caps, all of which may accelerate advanced plague formation in diabetes.

### Elevated SREBP-1 and IL-1β are functionally associated with the development of advanced lesions in diabetic, atherosclerotic pigs

**Figure 8:**
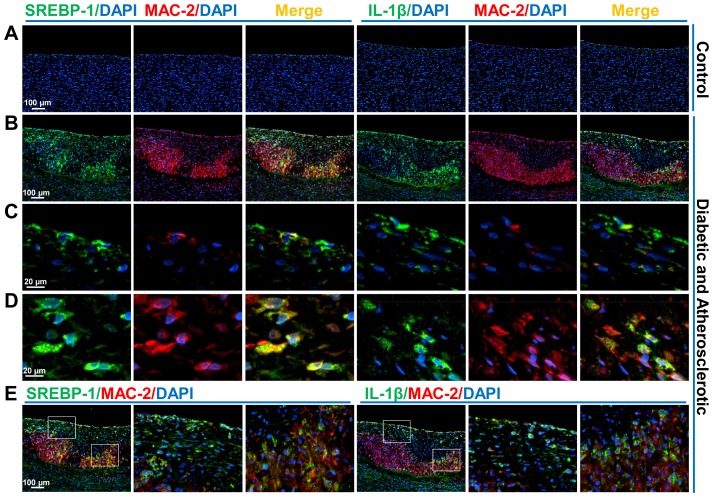
Immunofluorescent double staining for SREBP-1 and MAC-2 as well as for IL-1β and MAC-2 in aortic sections of control pigs and diabetic, atherosclerotic pigs. A representative immunofluorescence staining of adjacent aortic sections of control pigs (**A**) and diabetic, atherosclerotic pigs (**B–E**) is shown. Scale Bars: 100 µm or 20 µm. **A**. Immunofluorescent staining for SREBP-1 (green), IL-1β (green), or MAC-2 (red), and nuclear staining of DAPI (blue) in control pig aorta is shown. **B**. The staining for either SREBP-1 (left panel) or IL-1β (right panel) is detected in endothelial and smooth muscle layers as well as in MAC-2 positive macrophages of atheromatous plaques. **C**. positive cells of SREBP-1 or IL-1β are present in endothelial layers within lesions. **D**. Double staining-positive cells (yellow) either for SREBP-1 and MAC-2 or for IL-1β and MAC-2 are also located primarily in macrophage-rich regions within the subendothelial and intimal lesions. **E**. The regions representing an endothelium/intimal lesion area and a macrophage-rich core plaque area, respectively, are selected and presented as the enlarged images, so that the different cell types involved in the role of SREBP and IL-1β within lesions can be clearly observed.

Recent studies have identified that SREBP-1a is a major isoform in macrophages and directly upregulates the transcription of NLRP inflammasomes by its binding to the SRE element on NLRP promoters [11]. Real-time PCR showed that mRNA levels of SREBP-1a were substantially increased nearly 15-fold in the diabetic, atherosclerotic pig aorta (Fig. 6E), which was consistent with accumulation of macrophages in the lesions, as confirmed by robustly increased staining for the macrophage marker MAC-2 (a macrophage marker) (Fig. 8B–D). Consistent with other *in vitro* studies [11], elevated SREBP-1a is potentially involved in macrophage activation during atherogenesis *in vivo*.

To gain further insight into the cell types of the expression of SREBP-1 and IL-1β in atherosclerotic lesions, immunofluorescent double staining experiments for SREBP-1 and MAC-2 as well as for IL-1β and MAC-2 were performed in adjacent aortic sections of normal and atherosclerotic pigs. As shown in Fig. 8 and Fig. S1, immunofluorescent staining confirmed that SREBP-1 was obviously seen on the endothelium of intimal lesions (Fig. 8B, C, and E and Fig. S1A). This observation was consistent with the overlapping of SREBP-1 positive cells with eNOS-positive endothelial cells of lesions, as determined by immunohistochemical analysis (Fig. 7B and C). A robust increase in double positive staining for SREBP-1 and MAC-2 was also evident in macrophage-dense areas of intimal lesions and the subendothelial space (Fig. 8B, D, and E and Fig. S1A). Notably, positive staining for SREBP-1 was primarily located in the nuclei of some, but not all, of MAC-2-positive macrophages in lesions (Fig. 8D), which probably reflects different stages of SREBP-1 processing. Positive staining for MAC-2 was predominantly located in the cytoplasm of macrophages within subendothelial lesions (Fig. 8D). Intriguingly, strongly positive staining of IL-1β was also observed in the endothelial and smooth muscle layers of intimal lesions in atherosclerotic pigs, comparable to that of SREBP-1 (Fig. 8B, C and E and Fig. S1B), despite minimal staining for IL-1β in normal pigs (Fig. 8A and Fig. S1B). Immunofluorescent double staining indicated that the colocalization of IL-1β-positive cells and MAC-2-positive cells was present in primarily macrophage-enriched plaque areas of the subendothelial space (Fig. 8D), similarly to the distribution pattern of SREBP-1. These data indicate that inflammasome-mediated production of IL-1β is present not only in endothelial cells and smooth muscle cells of intimal lesions but also in macrophage-enriched lesion areas. Together with elevated staining for SREBP-1 and IL-1β in multiple cell types of intimal lesions, these results support the notion that abnormally enhanced SREBP-1 and NLRP3-IL-1β signaling contributes to the dysfunction of endothelial and smooth muscle cells and the activation of macrophages, ultimately leading to the acceleration of plaque formation.

## Discussion

**Figure 9:**
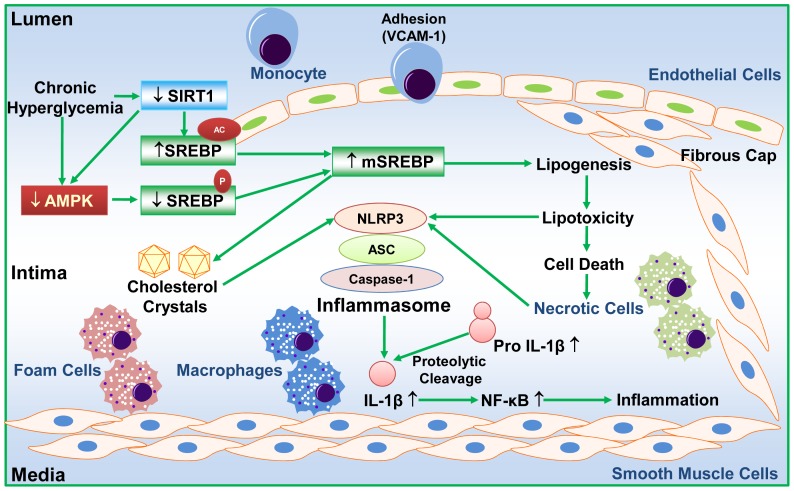
The proposed model for the association of SREBP-mediated lipotoxic signaling with NLRP3 inflammasome in advanced atherosclerotic plaques during diabetes. Prolonged hyperglycemia causes the integrated suppression of arterial SIRT1 and AMPK in the development of atherosclerosis. This coordinated dysregulation may contribute to the repression in SIRT1-mediated deacetylation of SREBP-1 and inhibition in AMPK-dependent Ser-372 phosphorylation of SREBP-1, thereby leading to the proteolytic activation of SREBP-1. Consequently, aberrant activation of SREBP stimulates expression of its target genes involved in *de novo* lipogenesis and cholesterol synthesis, promotes the formation of cholesterol crystals, and accelerates more lipid deposit in the arterial wall. On the other hand, the induction of the NLRP3 inflammasome, which is potentially triggered by sensing the toxic lipids, cholesterol crystals, and components of necrotic cells, possibly elicits caspase-1-dependent production of pro-atherogenic cytokine IL-1β. As a result, excess IL-1β production potentially activates vascular inflammatory response likely through the induction of NF-κB and its target genes such as VCAM-1 and iNOS, which ultimately causes eNOS reduction and vascular dysfunction. The inflammatory process may also promote the migration of smooth muscle cells from the media into the inflamed intima, contributing to the fibrous cap formation in advanced lesions. Collectively, activation of SREBP and elevation of NLRP3 inflammasome markers may explain much of complicated atherosclerotic characteristics, such as macrophage infiltration, endothelial cell dysfunction, smooth muscle cell migration and proliferation, deposition of cholesterol crystals, and formation of fibrous caps and necrotic cores.

The present study provides strong evidence that the integrated dysregulation of SIRT1-AMPK-SREBP pathway and elevation of NLRP3 inflammasome components in the vascular wall contribute significantly to early and advanced atherosclerotic development. The porcine model of diabetes and atherosclerosis exhibits complex lesion phenotypes including fatty streaks, fibrous caps, necrotic lipid cores, and deposition of cholesterol crystals. SREBP-1 positive cells are localized predominantly in eNOS-positive endothelial cells, MAC-2 positive macrophages, and VCAM-1-positive vascular cells within intimal lesions and cholesterol crystal-containing areas of advanced lesions. The improper SREBP activation in atherosclerotic pig aorta is evidenced by an increase in the proteolytic processing of SREBP-1 and -2 without significantly affecting their precursors, as well as an elevation of their target genes involved in *de novo* lipid synthesis. The induction of vascular lipid accumulation and inflammatory response is evidenced by the localization of either SREBP-1 or IL-1β in endothelial cells of inflamed intima and in MAC-2 positive macrophages of lipid-rich lesion areas, upregulation of NLRP3, ASC, and IL-1β, and activation of NF-κB-dependent genes, iNOS and COX-2. The pro-atherogenic effects of SREBP-1 and IL-1β may be implicated on endothelial cell dysfunction, macrophage activation, and smooth muscle cell proliferation within fibrous caps of advanced plaques. Moreover, SIRT1 and AMPK activities are substantially suppressed in atherosclerotic pig aorta. This dysregulation contributes to the aberrant activation of SREBP-1 probably through the impairment of deacetylation and phosphorylation of SREBP-1. Most importantly, the preclinical studies from diabetic individuals with coronary artery atherosclerosis also indicate that immunoreactivity of SREBP-1 is predominantly localized in VCAM-1-positive vascular cells and lipid-rich macrophages of the plaques. Although other factors such as the accumulation of oxidized LDL in macrophages have been implicated in the initiation of atherosclerosis [1], [2], the data presented here may suggest an attractive alternative model, in which the dysregulation of SIRT1-AMPK-SREBP signaling and elevation of NLRP3 inflammasome components result in vascular lipid deposit and elicit inflammatory process in atherosclerosis related to diabetes (Fig. 9).

### Vascular suppression of SIRT1 and AMPK plays a role in the stimulation of arterial SREBP-1 and excess lipid deposits in the development of atherosclerotic lesions

SIRT1 and AMPK, two important nutrient sensors, have been reported to exert multiple protective effects on vascular functions through the inhibition of inflammation, oxidant stress, vascular smooth muscle cell proliferation, and insulin resistance [36], [37]. Our studies and others have demonstrate that small molecule activators of SIRT1 or AMPK, such as resveratrol, the synthetic polyphenol S17834, and metformin, reduce the risk of hyperlipidemia for developing cardiovascular complications of obesity and type 2 diabetes [6], [19], [37]. We have recently shown that high-fat diet-induced obesity results in a decrease in the SIRT1-dependent deacetylation of lysine-382 on p53 and an elevation in apoptotic signaling in atherosclerotic lesion-prone aortic endothelium in mice [30]. Conversely, endothelium-specific overexpression of SIRT1 attenuates atherosclerotic lesions in ApoE^−/−^ mice [38]. The pro-atherogenic effect of AMPK deficiency is also emphasized by the fact that the loss of AMPKα2 accelerates aortic atherosclerotic development in LDLR^−/−^ mice [39]. An important finding of the present study is that *in vivo* concomitant inhibition of SIRT1 and AMPK is implicated in aortic atherosclerosis, since expression and activity of SIRT1 as well as phosphorylation of AMPK and its downstream targets ACC and SREBP-1 are remarkably suppressed in atherosclerotic pig aorta. The ability of SIRT1 to stimulate AMPK signaling is strongly supported by our previous observation that SIRT1 regulates hepatocyte lipid metabolism by activating AMPK [40] and by the Sinclair group showing that SIRT1 is required for AMPK activation and the beneficial effects of resveratrol on mitochondrial function *in vivo* [41]. How hyperglycemia and diabetes diminish the SIRT1-AMPK axis in atherosclerosis warrants further investigation.

Our previous studies indicate that inhibition of AMPK is responsible for sustained high glucose-induced SREBP-1 activation and lipid accumulation in cultured cells [6], [20], since SREBP-1 activation and triglyceride accumulation in cells exposed to high glucose are attenuated by the constitutively active AMPK and augmented by the dominant-negative AMPK [6]. Furthermore, molecular and biochemical evidence indicates that SREBP activity is downregulated by SIRT1 and AMPK via two distinct mechanisms including SIRT1-dependent deacetylation of SREBP and AMPK-dependent phosphorylation of SREBP [6], [31], [32]. An additional important finding is that an increase in the cleavage processing of SREBP-1 and -2 and a parallel elevation in their target genes such as FAS, SCD1, GPAT1, and HMGCS are observed in atherosclerotic pigs. Immunostaining clearly demonstrate that unlike SREBP-1 only detected in endothelial cells of normal pig aorta, positive staining for SREBP-1 in early and progressive plaques is more intense in activated endothelial cells and macrophages, but to a lesser extent in smooth muscle cells. The most likely explanation is that the pro-atherogenic effect of SREBP-1 appears vascular endothelial cell-specific or macrophage-specific. In support of our findings, SREBP activity in endothelial cells is previously shown to be increased by atherogenic stimuli such as shear stress and oxidized phospholipids [42], [43]. The present study provides the *in vivo* evidence that the upregulation of SREBP-1 may be a consequence of SIRT1 inhibition, which acts directly by diminishing SIRT1-mediated deacetylation of SREBP-1 and indirectly by repressing AMPK-mediated phosphorylation of SREBP-1 in atherosclerotic pigs, because pharmacological and genetic manipulation of SIRT1 can modulate AMPK signaling via LKB1 both *in vivo* and *in vitro* [40], [41], [44].

To determine whether SREBP is aberrantly altered in atherosclerosis, the study presented here suggests that SREBP likely plays a role in vascular lipid accumulation and inflammation during atherogenesis. First, SREBP-mediated lipotoxic signaling is elevated in the aortic atherosclerosis, which is possibly attributed to macrophage infiltration into the vascular wall of atherosclerosis. Second, the proteolytic processing of SREBP is highly active in atherosclerotic pig aorta. Because similar levels of SREBP-1 precursors are noted between control and atherosclerotic pig aorta, other regulators are possibly involved in the regulation of the proteolytic activation of SREBP during atherosclerosis. Further analysis also suggests that associated suppression of SIRT1 and AMPK may be implicated in impaired deacetylation and phosphorylation of SREBP-1, increased mature active form of SREBP-1, enhanced transcription of its target genes, and ultimately leads to excess lipid accumulation and deposit in arteries. Third, it raises the additional possibility that the abnormal activation of SREBP may ultimately lead to a potential vicious cycle of vascular lipid accumulation and inflammation in atherosclerosis. On account of the complexity of SREBP regulation, future studies will establish the mechanisms of cell type-specific effects of SREBP in atherosclerosis. Although we have established the role of SREBP in the porcine model of diabetes-accelerated atherosclerosis, which nearly resembles the onset of human atherosclerosis, activation of SREBP may occur in human atherosclerosis under conditions of hyperglycemia, hyperlipidemia, or both. Future clinical related studies are needed to investigate the impact of SREBP in atherosclerotic subjects with non-diabetes and diabetes.

### Stimulation of the NLRP3 inflammasome may contribute to vascular inflammatory response in the development of aortic atherosclerosis

While chronic and low-grade inflammation, an important pathogenic process of diabetes and atherosclerosis, can be elicited by metabolic signals, how nutrient stresses such as hyperglycemia and lipotoxicity initiate and sustain inflammation in atherosclerotic plaques is not well understood. Therefore, defining the molecular triggers and the cellular sensors that drive inflammation is of therapeutic importance. Recent studies indicate that the mice lacking components of NLRP3 inflammasome display anti-inflammatory and anti-atherosclerotic phenotypes [7]. These mice are also protected against insulin resistance and metabolic dysfunction during obesity-induced diabetes [8], depicting that the NLRP3 inflammasome may function as a sensor that detects danger signals and provokes inflammatory reaction in these diseases. Studies by Im et al. have discovered that SREBP-1a deficiency in macrophages diminishes LPS-induced activation of lipogenesis and NLRP inflammasomes [11]. This translational study clearly indicates the increase in SREBP-1 occurs in atherosclerotic lesions in both pigs and humans. Because expression of VCAM-1 reflects an inflammatory state in human atherosclerosis [1], [2], the clinical relevance of elevated SREBP-1 to vascular inflammation is evidenced by overlapped staining of SREBP-1 with VCAM-1 in atherosclerotic plaques in humans. These changes are associated with an increase in NLRP3 inflammasome signaling, co-localization of IL-1β and MAC-2 (a macrophage marker), and an elevation of NF-κB and its responsive genes iNOS and COX-2 in lesion areas, leading to initiated and amplified vascular inflammation. Cholesterol crystal-induced NLRP3 inflammasome is thought to be required for IL-1β secretion in macrophages and *in vivo* atherogenesis in mice [7]. The stimulation of SREBP-2-dependent cholesterol synthesis in atherosclerotic pig aorta may not only promote the formation of cholesterol crystals in advanced lesions but also potentially induce atherogenic inflammasomes. Considering that cells undergoing necrosis can also activate the NLRP3 inflammasome in macrophages [10], it is possible that several danger signals of inflammasomes, such as SREBP-1-mediated lipotoxic signaling, undissolved cholesterol crystals, and ATP released from necrotic cells, are involved in NLRP3-mediated induction of IL-1β in atherosclerosis. To our knowledge, these studies with atherosclerotic pigs provide the first *in vivo* evidence suggesting that dysfunctional SIRT1-AMPK-SREBP pathway appears to be involved in the formation of cholesterol crystals, which may account for the stimulation of NLRP3 inflammasome in atherosclerosis.

In conclusion, the present study uncovers a previously unrecognized role of SREBP in atherosclerosis and its functional association with NLRP3 inflammasome-driven inflammation. Such knowledge of improper SREBP-1 activity in human atherosclerosis has a potential prognostic and clinical implication for vascular lipid accumulation and inflammation. Because the integrated suppression of vascular SIRT1 and AMPK have an impact on activation of SREBP in arterial atherosclerosis, our findings highlight the rationale for targeting SIRT1-AMPK–SREBP signaling as a potential therapeutic treatment of human atherosclerosis.

## Supporting Information

Figure S1 **A representative immunofluorescence double staining of adjacent aortic sections of control pigs and diabetic, atherosclerotic pigs is shown.** **A**. Immunofluorescent double staining for SREBP-1 (green) and MAC-2 (red), nuclear staining with DAPI (blue), and merging images are shown. **B**. Immunofluorescent double staining for IL-1β (green), MAC-2 (red), nuclear staining (blue), and merging images are shown. The regions representing an endothelium/intimal lesion area and a macrophage-rich core plaque area, respectively, are selected and presented as the enlarged images, so that the different cell types involved in the role of SREBP-1 and IL-1β can be obviously observed. Scale Bars: 100 µm or 50 µm.
(TIF)

## References

[1] Libby P, Ridker PM, Hansson GK (2011) Progress and challenges in translating the biology of atherosclerosis. Nature 473: 317–325. 21593864 10.1038/nature10146

[2] Bornfeldt KE, Tabas I (2011) Insulin Resistance, Hyperglycemia, and Atherosclerosis. Cell Metabolism 14: 575–585. 22055501 10.1016/j.cmet.2011.07.015 PMC3217209

[3] Goldstein JL, Brown MS (2008) From fatty streak to fatty liver: 33 years of joint publications in the JCI. Journal of Clinical Investigation 118: 1220–1222. 18382725 10.1172/JCI34973 PMC2276798

[4] Raghow R, Yellaturu C, Deng X, Park EA, Elam MB (2008) SREBPs: the crossroads of physiological and pathological lipid homeostasis. Trends in Endocrinology and Metabolism 19: 65–73. 18291668 10.1016/j.tem.2007.10.009

[5] Horton JD, Goldstein JL, Brown MS (2002) SREBPs: activators of the complete program of cholesterol and fatty acid synthesis in the liver. Journal of Clinical Investigation 109: 1125–1131. 11994399 10.1172/JCI15593 PMC150968

[6] Li Y, Xu SQ, Mihaylova MM, Zheng B, Hou XY, et al (2011) AMPK Phosphorylates and Inhibits SREBP Activity to Attenuate Hepatic Steatosis and Atherosclerosis in Diet-Induced Insulin-Resistant Mice. Cell Metabolism 13: 376–388. 21459323 10.1016/j.cmet.2011.03.009 PMC3086578

[7] Duewell P, Kono H, Rayner KJ, Sirois CM, Vladimer G, et al (2010) NLRP3 inflammasomes are required for atherogenesis and activated by cholesterol crystals. Nature 464: 1357–13U7. 20428172 10.1038/nature08938 PMC2946640

[8] Vandanmagsar B, Youm YH, Ravussin A, Galgani JE, Stadler K, et al (2011) The NLRP3 inflammasome instigates obesity-induced inflammation and insulin resistance. Nature Medicine 17: 179–U214. 10.1038/nm.2279 PMC3076025 21217695

[9] Schroder K, Tschopp J (2010) The Inflammasomes. Cell 140: 821–832. 20303873 10.1016/j.cell.2010.01.040

[10] Iyer SS, Pulskens WP, Sadler JJ, Butter LM, Teske GJ, et al (2009) Necrotic cells trigger a sterile inflammatory response through the Nlrp3 inflammasome. Proceedings of the National Academy of Sciences of the United States of America 106: 20388–20393. 19918053 10.1073/pnas.0908698106 PMC2787135

[11] Im SS, Yousef L, Blaschitz C, Liu JZ, Edwards RA, et al (2011) Linking Lipid Metabolism to the Innate Immune Response in Macrophages through Sterol Regulatory Element Binding Protein-1a. Cell Metabolism 13: 540–549. 21531336 10.1016/j.cmet.2011.04.001 PMC3090630

[12] Jawien J, Nastalek P, Korbut R (2004) Mouse models of experimental atherosclerosis. Journal of Physiology and Pharmacology 55: 503–517. 15381823

[13] Gerrity RG, Natarajan R, Nadler JL, Kimsey T (2001) Diabetes-induced accelerated atherosclerosis in swine. Diabetes 50: 1654–1665. 11423488 10.2337/diabetes.50.7.1654

[14] Xu S, Ying J, Jiang B, Guo W, Adachi T, et al (2006) Detection of sequence-specific tyrosine nitration of manganese SOD and SERCA in cardiovascular disease and aging. Am J Physiol Heart Circ Physiol 290: H2220–H2227. 16399855 10.1152/ajpheart.01293.2005

[15] Li Y, Xu SQ, Giles A, Nakamura K, Lee JW, et al (2011) Hepatic overexpression of SIRT1 in mice attenuates endoplasmic reticulum stress and insulin resistance in the liver. Faseb Journal 25: 1664–1679. 21321189 10.1096/fj.10-173492 PMC3079300

[16] Zuccollo A, Shi CM, Mastroianni R, Maitland-Toolan KA, Weisbrod RM, et al (2005) The thromboxane A(2) receptor antagonist S18886 prevents enhanced atherogenesis caused by diabetes mellitus. Circulation 112: 3001–3008. 16260636 10.1161/CIRCULATIONAHA.105.581892

[17] Gerrity RG, Naito HK, Richardson M, Schwartz CJ (1979) Dietary Induced Atherogenesis in Swine - Morphology of the Intima in Pre-Lesion Stages. American Journal of Pathology 95: 775-&. 453335 PMC2042303

[18] Ying J, Sharov V, Xu SQ, Jiang BB, Gerrity R, et al (2008) Cysteine-674 oxidation and degradation of sarcoplasmic reticulum Ca(2+) ATPase in diabetic pig aorta. Free Radical Biology and Medicine 45: 756–762. 18590812 10.1016/j.freeradbiomed.2008.05.029 PMC2654240

[19] Zang MW, Xu SQ, Maitland-Toolan KA, Zuccollo A, Hou XY, et al (2006) Polyphenols stimulate AMP-activated protein kinase, lower lipids, and inhibit accelerated atherosclerosis in diabetic LDL receptor-deficient mice. Diabetes 55: 2180–2191. 16873680 10.2337/db05-1188

[20] Zang MW, Zuccollo A, Hou XY, Nagata D, Walsh K, et al (2004) AMP-activated protein kinase is required for the lipid-lowering effect of metformin in insulin-resistant human HepG2 cells. Journal of Biological Chemistry 279: 47898–47905. 15371448 10.1074/jbc.M408149200

[21] Libby P, Theroux P (2005) Pathophysiology of coronary artery disease. Circulation 111: 3481–3488. 15983262 10.1161/CIRCULATIONAHA.105.537878

[22] Foretz M, Pacot C, Dugail I, Lemarchand P, Guichard C, et al (1999) ADD1/SREBP-1c is required in the activation of hepatic lipogenic gene expression by glucose. Molecular and Cellular Biology 19: 3760–3768. 10207099 10.1128/mcb.19.5.3760 PMC84202

[23] Vaziri H, Dessain SK, Eagon EN, Imai SI, Frye RA, et al (2001) hSIR2(SIRT1) functions as an NAD-dependent p53 deacetylase. Cell 107: 149–159. 11672523 10.1016/s0092-8674(01)00527-x

[24] Cheng HL, Mostoslavsky R, Saito S, Manis JP, Gu Y, et al (2003) Developmental defects and p53 hyperacetylation in Sir2 homolog (SIRT1)-deficient mice. Proc Natl Acad Sci U S A 100: 10794–10799. 12960381 10.1073/pnas.1934713100 PMC196882

[25] Chua KF, Mostoslavsky R, Lombard DB, Pang WW, Saito S, et al (2005) Mammalian SIRT1 limits replicative life span in response to chronic genotoxic stress. Cell Metabolism 2: 67–76. 16054100 10.1016/j.cmet.2005.06.007

[26] Howitz KT, Bitterman KJ, Cohen HY, Lamming DW, Lavu S, et al (2003) Small molecule activators of sirtuins extend Saccharomyces cerevisiae lifespan. Nature 425: 191–196. 12939617 10.1038/nature01960

[27] Luo J, Nikolaev AY, Imai S, Chen D, Su F, et al (2001) Negative control of p53 by Sir2alpha promotes cell survival under stress. Cell 107: 137–148. 11672522 10.1016/s0092-8674(01)00524-4

[28] Luo JY, Su F, Chen DL, Shiloh A, Gu W (2000) Deacetylation of p53 modulates its effect on cell growth and apoptosis. Nature 408: 377–381. 11099047 10.1038/35042612

[29] Yin HQ, Ajmo JM, Murr M, You M (2012) Mir-217 is a Major Inflammatory Regulator in Experimental Alcoholic Steatohepatitis. Hepatology 56: 1119A.

[30] Xu S, Jiang B, Hou X, Shi C, Bachschmid MM, et al (2011) High-fat diet increases and the polyphenol, S17834, decreases acetylation of the sirtuin-1-dependent lysine-382 on p53 and apoptotic signaling in atherosclerotic lesion-prone aortic endothelium of normal mice. J Cardiovasc Pharmacol 58: 263–271 10.1097/FJC.0b013e3182239eb7 [doi]. 21654327 10.1097/FJC.0b013e3182239eb7 PMC3168693

[31] Ponugoti B, Kim DH, Xiao Z, Smith Z, Miao J, et al (2010) SIRT1 Deacetylates and Inhibits SREBP-1C Activity in Regulation of Hepatic Lipid Metabolism. Journal of Biological Chemistry 285: 33959–33970. 20817729 10.1074/jbc.M110.122978 PMC2962496

[32] Walker AK, Yang FJ, Jiang KR, Ji JY, Watts JL, et al (2010) Conserved role of SIRT1 orthologs in fasting-dependent inhibition of the lipid/cholesterol regulator SREBP. Genes & Development 24: 1403–1417. 20595232 10.1101/gad.1901210 PMC2895199

[33] You M, Liang XM, Ajmo JM, Ness GC (2008) Involvement of mammalian sirtuin 1 in the action of ethanol in the liver. American Journal of Physiology-Gastrointestinal and Liver Physiology 294: G892–G898. 18239056 10.1152/ajpgi.00575.2007

[34] Hansson GK (2005) Inflammation, atherosclerosis, and coronary artery disease - Reply. New England Journal of Medicine 353: 429–430. 16050062

[35] Goldin A, Beckman JA, Schmidt AM, Creager MA (2006) Advanced glycation end products - Sparking the development of diabetic vascular injury. Circulation 114: 597–605. 16894049 10.1161/CIRCULATIONAHA.106.621854

[36] Stein S, Matter CM (2011) Protective roles of SIRT1 in atherosclerosis. Cell Cycle 10: 640–647. 21293192 10.4161/cc.10.4.14863

[37] Towler MC, Hardie DG (2007) AMP-activated protein kinase in metabolic control and insulin signaling. Circulation Research 100: 328–341. 17307971 10.1161/01.RES.0000256090.42690.05

[38] Zhang QJ, Wang Z, Chen HZ, Zhou S, Zheng W, et al (2008) Endothelium-specific overexpression of class III deacetylase SIRT1 decreases atherosclerosis in apolipoprotein E-deficient mice. Cardiovascular Research 80: 191–199. 18689793 10.1093/cvr/cvn224 PMC3657473

[39] Dong YZ, Zhang M, Liang B, Xie ZL, Zhao ZX, et al (2010) Reduction of AMP-Activated Protein Kinase alpha 2 Increases Endoplasmic Reticulum Stress and Atherosclerosis In Vivo. Circulation 121: 792–803. 20124121 10.1161/CIRCULATIONAHA.109.900928 PMC2825900

[40] Hou X, Xu S, Maitland-Toolan KA, Sato K, Jiang B, et al (2008) SIRT1 regulates hepatocyte lipid metabolism through activating AMP-activated protein kinase. J Biol Chem 283: 20015–20026. 18482975 10.1074/jbc.M802187200 PMC2459285

[41] Price NL, Gomes AP, Ling AJ, Duarte FV, Martin-Montalvo A, et al (2012) SIRT1 Is Required for AMPK Activation and the Beneficial Effects of Resveratrol on Mitochondrial Function. Cell Metab 15: 675–690 S1550-4131(12)00143-X [pii];10.1016/j.cmet.2012.04.003 [doi]. 22560220 10.1016/j.cmet.2012.04.003 PMC3545644

[42] Lin T, Zeng LF, Liu Y, Defea K, Schwartz MA, et al (2003) Rho-ROCK-LIMK-cofilin pathway regulates shear stress activation of sterol regulatory element binding proteins. Circulation Research 92: 1296–1304. 12775580 10.1161/01.RES.0000078780.65824.8B

[43] Yeh M, Cole AL, Choi J, Liu Y, Tulchinsky D, et al (2004) Role for sterol regulatory element-binding protein in activation of endothelial cells by phospholipid oxidation products. Circulation Research 95: 780–788. 15388640 10.1161/01.RES.0000146030.53089.18

[44] Lan F, Cacicedo JM, Ruderman N, Ido Y (2008) SIRT1 modulation of the acetylation status, cytosolic localization, and activity of LKB1. Possible role in AMP-activated protein kinase activation. J Biol Chem 283: 27628–27635. 18687677 10.1074/jbc.M805711200 PMC2562073

