# Supplementary figures and images for: Activation of Sterol Regulatory Element Binding Protein and NLRP3 Inflammasome in Atherosclerotic Lesion Development in Diabetic Pigs
Source: PLoS One. 2013 Jun 25;8(6):e67532. doi: 10.1371/journal.pone.0067532 (PMC3692453; doi:10.1371/journal.pone.0067532)

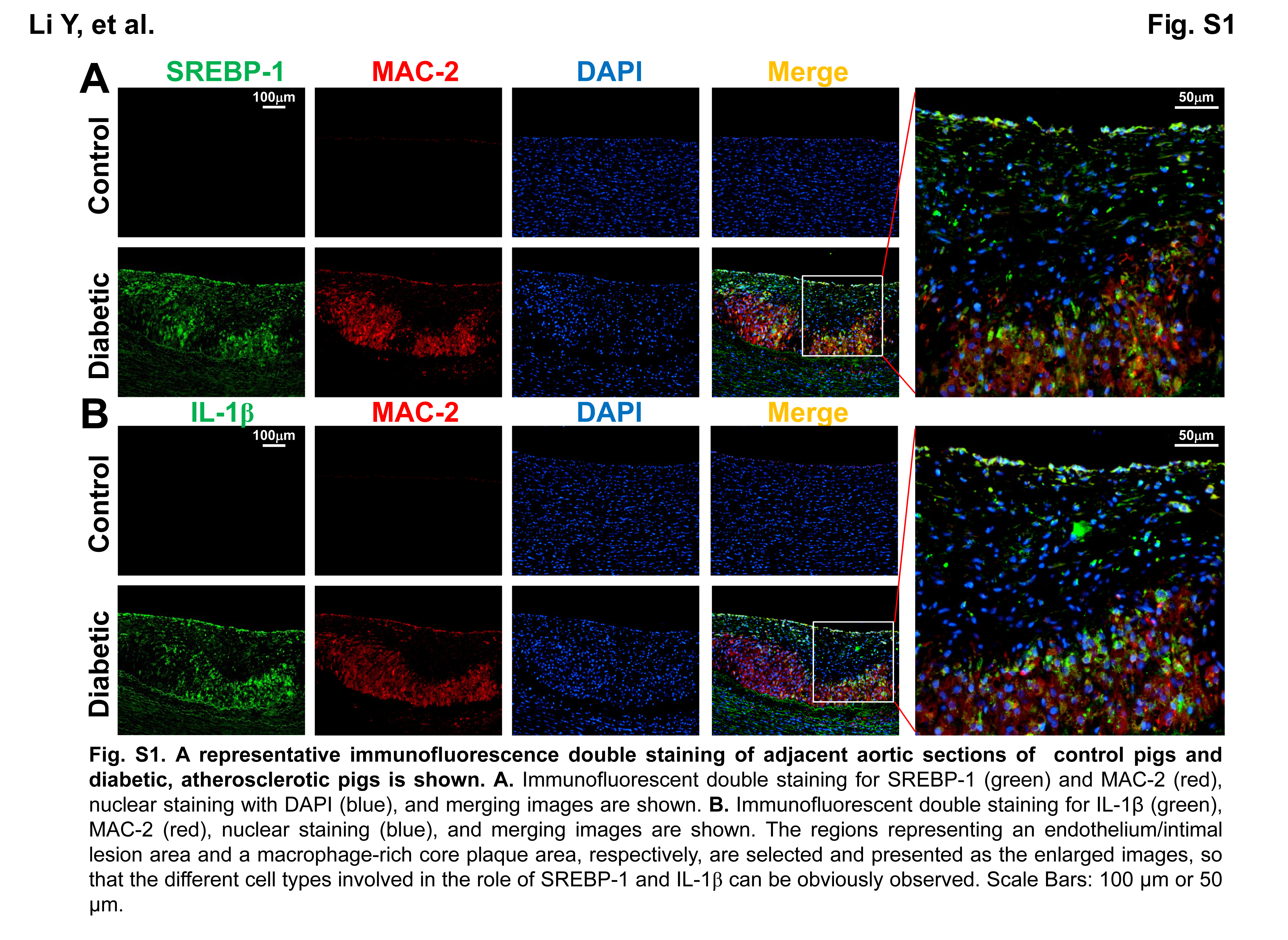

Supplement: Figure S1 — A representative immunofluorescence double staining of adjacent aortic sections of control pigs and diabetic, atherosclerotic pigs is shown. A. Immunofluorescent double staining for SREBP-1 (green) and MAC-2 (red), nuclear staining with DAPI (blue), and merging images are shown. B. Immunofluorescent double staining for IL-1β (green), MAC-2 (red), nuclear staining (blue), and merging images are shown. The regions representing an endothelium/intimal lesion area and a macrophage-rich core plaque area, respectively, are selected and presented as the enlarged images, so that the different cell types involved in the role of SREBP-1 and IL-1β can be obviously observed. Scale Bars: 100 µm or 50 µm. (TIF) [file pone.0067532.s001.tif]
